# Perinatal risk assessment in pregnancies complicated by early‐onset fetal growth restriction: development and internal validation of a prediction model for composite adverse perinatal outcome

**DOI:** 10.1002/uog.29265

**Published:** 2025-07-07

**Authors:** M. van de Meent, E. W. Bel, W. Ganzevoort, S. J. Gordijn, F. Groenendaal, E. M. W. Kooi, W. Onland, E. Schuit, A. T. Lely, J. Kooiman, on behalf of the OPtimal TIming of antenatal COrticosteroids in pregnancies complicated by early‐onset fetal growth REstriction (OPTICORE) study group

**Affiliations:** ^1^ Department of Obstetrics and Gynaecology University Medical Center Utrecht Utrecht The Netherlands; ^2^ Department of Obstetrics and Gynaecology Amsterdam University Medical Centers, location AMC Amsterdam The Netherlands; ^3^ Amsterdam Reproduction and Development Research Institute Amsterdam The Netherlands; ^4^ Department of Obstetrics and Gynaecology, University Medical Center Groningen University of Groningen Groningen The Netherlands; ^5^ Department of Neonatology, Wilhelmina Children's Hospital, University Medical Center Utrecht Utrecht University Utrecht The Netherlands; ^6^ Division of Neonatology, Beatrix Children's Hospital, University Medical Center Groningen University of Groningen Groningen The Netherlands; ^7^ Department of Neonatology Amsterdam University Medical Centers location AMC, Amsterdam The Netherlands; ^8^ Julius Center for Health Sciences and Primary Care, University Medical Center Utrecht Utrecht University Utrecht The Netherlands; ^9^ Department of Obstetrics and Gynaecology Erasmus Medical Center Rotterdam The Netherlands

**Keywords:** adverse perinatal outcome, clinical decision‐making, early‐onset fetal growth restriction, parental counseling, prediction model

## Abstract

**Objective:**

Early‐onset fetal growth restriction (FGR) frequently requires iatrogenic preterm delivery to prevent stillbirth or the sequelae of hypoxia. A prediction model for adverse perinatal outcome could aid clinical decision‐making and parental counseling. However, an adequate externally validated model, including predictors that are readily available for this purpose, is currently lacking. The aim of the present study was to develop a prediction model for composite adverse perinatal outcome (CAPO) to be used at the time of hospital admission for fetal monitoring in early‐onset FGR.

**Methods:**

A model was developed to predict CAPOs (defined as one or more of the following: perinatal or in‐hospital mortality, necrotizing enterocolitis ≥ Stage IIA, moderate or severe bronchopulmonary dysplasia, cystic periventricular leukomalacia, intraventricular hemorrhage ≥ Grade III or venous infarction and/or culture‐proven sepsis) in early‐onset FGR. The model was developed and internally validated in the OPTICORE study, a retrospective, multicenter cohort study of pregnancies complicated by early‐onset FGR, in accordance with the consensus‐based definition, in six academic hospitals in The Netherlands. Candidate predictors were selected based on the existing literature and expert opinion and comprised maternal medical history, obstetric history, fetal growth and Doppler assessment. A backward stepwise elimination procedure was performed based on the Aikake Information Criterion. Internal validation was performed by bootstrapping and repeating the predictor selection process to determine the shrinkage factor to adjust for overfitting. Internal–external cross‐validation was performed as a sensitivity analysis to assess the impact of clustering of patients within each hospital.

**Results:**

In total, 560/1453 (38.5%) pregnancies were complicated by CAPO. The developed model included 14 predicting variables, determined at the time of hospital admission for fetal monitoring: maternal history of chronic kidney disease or chronic hypertension, smoker, previous pregnancy complicated by FGR, gestational age at admission, fetal sex, concomitant pre‐eclampsia, the use of magnesium sulfate, gestational diabetes mellitus, estimated fetal weight, umbilical artery and middle cerebral artery pulsatility index percentile, absent or reversed end‐diastolic flow in the umbilical artery and gestational age at diagnosis of FGR. After internal validation and shrinkage to adjust for optimism, the model performed well (area under the receiver‐operating‐characteristics curve, 0.83 (95% CI, 0.79–0.87); calibration slope, 1.05 (95% CI, 0.94–1.17); calibration‐in‐the‐large, 0.07 (95% CI, −0.06 to 0.20)). The internal–external cross‐validation sensitivity analysis revealed equivalent model performance measures across the three largest hospitals.

**Conclusions:**

The developed model, including 14 readily available predictors, showed good performance for the prediction of CAPO at the time of hospital admission and may serve as a helpful tool for clinical decision‐making and parental counseling in the setting of early‐onset FGR. External validation and assessment of the model's impact on clinical decision‐making and patient outcomes are required before it can be implemented in routine clinical practice. © 2025 The Author(s). *Ultrasound in Obstetrics & Gynecology* published by John Wiley & Sons Ltd on behalf of International Society of Ultrasound in Obstetrics and Gynecology.

## INTRODUCTION

Early‐onset fetal growth restriction (FGR) is defined as the failure of a fetus to reach its growth potential (diagnosed < 32 weeks' gestation) and affects 0.5–1.0% of pregnancies^1^. It is primarily caused by placental dysfunction, leading to unmet fetal metabolic and gaseous demands. To prevent stillbirth or the sequelae of hypoxia, early‐onset FGR frequently requires iatrogenic preterm birth. Concomitant pre‐eclampsia (PE) necessitates preterm delivery in 30–40% of early‐onset FGR pregnancies^2^. Consequently, early‐onset FGR is a significant cause of perinatal morbidity (24% of FGR cases) and mortality (8–19% of FGR cases)^1^, ^3^, ^4^, ^5^, ^6^, ^7^.

Prediction models can aid clinical decision‐making by providing risk estimates of adverse perinatal outcomes based on information available at hospital admission for fetal monitoring, and aid individualized counseling and management of parental expectations following preterm or term birth in early‐onset FGR^8^. Additionally, in some countries, such as The Netherlands, expectant parents receive joint counseling from an obstetrician and a neonatologist regarding the course of the pregnancy and its likely trajectory in the neonatal intensive care unit after birth. If counseling is provided between 24 and 26 weeks' gestation and estimated fetal weight exceeds 500 g, parents can decide whether to opt for active fetal and subsequent neonatal management according to national protocol in The Netherlands. If gestational age exceeds 26 weeks and estimated fetal weight exceeds 500 g at the time of counseling, active fetal monitoring is the gold standard^9^. The prediction of neonatal outcomes could facilitate such counseling and joint decision‐making.

Several models have been developed to predict adverse perinatal outcomes in FGR, but most of these have been hampered by methodological issues, including missing information that is required for the performance of external validation in the majority of developed models, different moments of prediction, different definitions of FGR and predicted outcomes, and inclusion of biomarkers that are not readily available in every setting^10^, ^11^, ^12^.

Therefore, the aim of this study was to develop and internally validate a model to predict composite adverse perinatal outcome (CAPO) at the time of hospital admission to offer personalized perinatal risk assessment, which could aid clinical decision‐making and parental counseling in early‐onset FGR.

## METHODS

### Study design

We conducted a *post*‐*hoc* analysis of the OPtimal TIming of antenatal COrticosteroid administration in early‐onset fetal growth REstriction (OPTICORE) study^13^, ^14^, which is a multicenter, retrospective cohort study performed in six tertiary hospitals (Amsterdam University Medical Center, Amsterdam; Erasmus Medical Center, Rotterdam; Isala hospital, Zwolle; Maastricht University Medical Center, Maastricht; University Medical Center Groningen, Groningen; and University Medical Center Utrecht, Utrecht) in The Netherlands between January 2012 and December 2021 that aimed to compare the two main timing strategies of antenatal corticosteroid administration in early‐onset FGR in The Netherlands using practice variation. Patients were considered eligible for inclusion if they met all the following criteria: (1) at least 18 years of age; (2) singleton pregnancy; (3) early‐onset FGR diagnosis in accordance with the consensus‐based definition of Gordijn *et al*.^15^; and (4) consented to active fetal management. Exclusion criteria were multiple pregnancy, fetal congenital abnormality or antenatally diagnosed genetic disorder and patient refusal of the use of their data for scientific research. Patient data of the mother and their offspring were extracted from medical records. Outcome data were subsequently cross‐validated with the Dutch birth registry^16^. The methods followed in the OPTICORE study, including definitions of predictors and outcome variables, have been described extensively in the study protocol, published in 2023^13^. In The Netherlands, early‐onset FGR pregnancies are hospitalized from an early disease stage (hospitalization based on umbilical artery (UA) pulsatility index (PI) > 95^th^ percentile or absent or reversed end‐diastolic flow (A/REDF) in the UA) for daily cardiotocography (CTG) monitoring, weekly Doppler measurements and biweekly fetal growth scans. For those with UA‐A/REDF, CTG monitoring is increased to twice daily and Doppler measurements are increased to twice a week. Ductus venosus measurements were recorded, although this is not routine practice in most hospitals. The results of this study are reported in accordance with the Transparent Reporting of multivariable prediction model for Individual Prognosis or Diagnosis (TRIPOD) guidelines (Appendix S1)^17^.

### Candidate predictors

Candidate predictors were selected based on the existing literature and clinical reasoning by experts in the field (Table 1)^12^. Since estimated fetal weight, gestational age at admission and maternal history of chronic hypertension were determined to be significant predictors of CAPO in previous FGR studies, these candidate predictors were forced into the model during its development^12^. Using a CAPO rate of 31.2%, as reported in the landmark FGR Trial of Randomized Umbilical and Fetal Flow in Europe (TRUFFLE) study, a c‐statistic of 0.75 and a sample size of 1453, the maximum number of candidate predictors that could be considered for model development was 28^4^, ^18^. We considered 27 relevant candidate predictors for model development.

**Table 1:** Overview of 27 candidate predictors, classified as maternal baseline characteristics, medical history and obstetric history, current pregnancy parameters, fetal ultrasound parameters and fixed parameters

Phase	Candidate predictor
Maternal baseline characteristics, medical history and obstetric history (Phase A)	Age BMI Smoker Chronic kidney disease APS/SLE Parous Previous FGR Previous preterm birth Previous PE/HELLP syndrome Previous perinatal mortality Previous gestational diabetes mellitus
Current pregnancy parameters (Phase B)	Gestational age at FGR diagnosis Fetal sex PIH PE/HELLP syndrome Use of antihypertensive drugs Hypertensive crisis Use of magnesium sulfate (maternal and/or fetal indication) Gestational diabetes mellitus Use of antenatal CCS at any time during pregnancy
Fetal ultrasound parameters (Phase C)	UA‐PI percentile MCA‐PI percentile Absent or reversed end‐diastolic flow in the UA Cerebroplacental ratio percentile
Fixed parameters	Chronic hypertension Gestational age at admission Estimated fetal weight

APS, antiphospholipid syndrome; BMI, body mass index; CCS, corticosteroids; FGR, fetal growth restriction; HELLP, Hemolysis, Elevated Liver enzymes, and Low Platelet; MCA, middle cerebral artery; PE, pre‐eclampsia; PI, pulsatility index; PIH, pregnancy‐induced hypertension; SLE, systemic lupus erythematosus; UA, umbilical artery.

### Outcome

The predicted outcome was in accordance with the TRUFFLE study and defined as a composite of at least one of the following adverse perinatal outcome measures before hospital discharge: intraventricular hemorrhage ≥ Grade III or venous infarction; culture‐proven sepsis; necrotizing enterocolitis ≥ Stage IIA; cystic periventricular leukomalacia; moderate or severe bronchopulmonary dysplasia; and/or perinatal or in‐hospital mortality^4^. Definitions of these outcomes are provided in Table S1, ^13^.

### Data quality and missing data

The prognosis was determined at the time of first hospital admission beyond the fetal viability level (> 24 weeks in The Netherlands), with the indication for admission based on local protocol. Data regarding predictor variables were collected for the period within 48 h after admission. If not available, the last measurement taken within 2 weeks before admission was regarded as the measurement at hospital admission (i.e. last observation was carried forward), in accordance with clinical practice and a previously developed prediction model in pregnancies complicated by PE (the fullPIERS model)^19^. For UA‐PI, middle cerebral artery (MCA)‐PI and the cerebroplacental ratio, percentiles were calculated for the corresponding gestational age based on previously described reference ranges^20^, ^21^, ^22^. The proportion of missing outcome data was minimal, with 6.1% missing for bronchopulmonary dysplasia, while complete data were available for the remaining outcomes. Assuming that missing data were missing at random, multiple imputation was performed for missing data on the exposure, predictors and outcome using the ‘MICE’ package, to prevent loss of precision and the introduction of bias^23^, ^24^, ^25^. The imputation model included all candidate predictors and all components of CAPO. The number of imputations required for accurate point estimates and standard errors was determined using the ‘howManyImputations’ package (R Foundation for Statistical Computing, Vienna, Austria) (*n* = 165)^26^.

### Statistical analysis

Baseline characteristics were analyzed using descriptive statistics. Values are provided as mean ± SD or median (interquartile range), as appropriate, for continuous variables and as *n* (%) for dichotomous variables across the imputed datasets. The linearity of the association between continuous variables and CAPO was assessed using multivariable fractional polynomials^27^. In case of non‐linearity, continuous predictor variables were transformed as appropriate.

First, univariable logistic regression analyses were performed to determine the association between each individual variable and CAPO, and results were presented as odds ratios (OR) with 95% CIs. Multivariable logistic regression analyses were performed to build the final prediction model. All candidate predictors were included in the multivariable analyses, as the selection of predictors based solely on univariable analysis can result in unstable prediction models^28^, ^29^. The selection of predictors, apart from the three fixed predictors, was performed using backward stepwise selection in three phases, in the order in which the parameters became available during patient consultation at hospital admission (e.g. the predictors remaining after backward stepwise selection in Phase A were supplemented with predictors from Phase B, on which the selection procedure was carried out again): Phase A, maternal baseline characteristics, medical history and obstetric history; Phase B, current pregnancy parameters; and Phase C, fetal ultrasound parameters. Backward stepwise selection was applied to select the final set of predictors based on the Aikake Information Criterion. The predictor selection process was performed for all 165 imputed datasets and a majority rule was applied (i.e. the final set of predictors included those that were selected in the majority of datasets)^30^.

Model calibration was assessed by visual inspection of the calibration plots and numerically by determination of the calibration slope (ideal value = 1) and calibration‐in‐the‐large (ideal value = 0). For the calibration plots, patients were ranked by estimated probability, divided into ten groups and the average predicted probability and average observed outcome were subsequently determined. Model discrimination was reported as the area under the receiver‐operating‐characteristics curve (AUC) (ideal value = 1). The intercepts, coefficients and model performance parameters of the final model were pooled across imputed datasets using Rubin's rule^31^. Internal validation was performed using 1000 bootstrap samples in each of the imputed datasets. Within each bootstrap sample, the predictor selection process was repeated to determine the shrinkage factor to be multiplied with all the regression coefficients to adjust for overfitting^29^. After shrinkage, the model's intercept was re‐estimated using an offset model.

As a sensitivity analysis, model development was repeated by internal–external cross‐validation (i.e. development of the model in five hospitals and external validation in the remaining hospital, with each hospital serving as the external validation set once)^32^. Afterwards, model performance measures on external validation were pooled across hospitals using random‐effects meta‐analysis, and heterogeneity in performance measures was assessed using the *I* ^2^ statistic. Using a rate of 31.2%, a mean linear predictor of −0.843 and a SD of 1.660, the required sample size for external validation was determined to be 1816 (848 for the observed *vs* expected ratio, 1816 for the calibration slope and 352 for the AUC)^33^. Since the required sample size was not achieved in any of the included hospitals, the internal–external cross‐validation results serve solely as an impression of the consistency of performance of the model across hospitals. Statistical analysis was performed in R version 4.3.2 (R Foundation)^34^.

## RESULTS

Between 2012 and 2021, a total of 1453 pregnancies were included in the OPTICORE cohort^13^. Maternal demographic and baseline characteristics, and neonatal characteristics before multiple imputation (Tables S2 and S3) and after multiple imputation (Tables 2 and 3), are summarized. Of the 1453 pregnancies, 560 (38.5%) were complicated by CAPO (Table 3). Univariable analysis showed an increased risk of CAPO associated with concomitant PE/HELLP (Hemolysis, Elevated Liver enzymes, and Low Platelet) syndrome (OR, 1.79 (95% CI, 1.44–2.22)), use of antihypertensive drugs (OR, 1.57 (95% CI, 1.25–1.96)), use of magnesium sulfate (OR, 1.96 (95% CI, 1.52–2.54)) and UA‐A/REDF (OR, 2.52 (95% CI, 1.99–3.20)), and a decreased risk of CAPO associated with higher estimated fetal weight (OR, 0.51 (95% CI, 0.46–0.58)) and a higher gestational age at admission (OR, 0.93 (95% CI, 0.92–0.94)) and FGR diagnosis (OR, 0.73 (95% CI, 0.69–0.78)) (Tables 2 and S4).

**Table 2:** Demographic and baseline characteristics at hospital admission in pregnancies with and those without composite adverse pregnancy outcome (CAPO), after multiple imputation

Parameter	Without CAPO (*n* = 893)	With CAPO (*n* = 560)	Overall (*n* = 1453)	*P* *
Maternal age (years)	30.6 ± 5.5	30.6 ± 5.4	30.6 ± 5.4	0.95
Maternal BMI (kg/m^2^)	24.4 (20.7–30.1)	24.8 (21.3–30.5)	24.6 (20.9–30.2)	0.08
White maternal ethnicity	613 (68.7)	391 (69.8)	1004 (69.1)	0.29
Medical history				
Chronic hypertension	103 (11.5)	100 (17.9)	203 (14.0)	< 0.01
Chronic kidney disease	27 (3.1)	20 (3.5)	47 (3.2)	0.79
SLE/APS	8 (0.9)	8 (1.4)	16 (1.1)	0.49
Pre‐existing diabetes mellitus	17 (1.9)	15 (2.7)	32 (2.2)	0.43
Smoker during pregnancy	160 (18.0)	71 (12.6)	231 (15.9)	< 0.01
Obstetric history				
Parous	327 (36.6)	198 (35.3)	525 (36.1)	0.71
Previous FGR	90 (10.1)	75 (13.3)	165 (11.4)	0.11
Previous PIH	19 (2.1)	21 (3.7)	40 (2.7)	0.07
Previous PE/HELLP syndrome	61 (6.8)	62 (11.0)	122 (8.4)	< 0.01
Previous preterm birth	122 (13.6)	88 (15.7)	210 (14.4)	0.33
Previous perinatal mortality	40 (4.5)	26 (4.7)	66 (4.5)	0.85
Previous gestational diabetes mellitus	17 (2.0)	8 (1.4)	25 (1.7)	0.96
Current pregnancy				
PIH	141 (15.8)	84 (14.9)	227 (15.6)	0.88
PE/HELLP syndrome	281 (31.4)	252 (45.0)	533 (36.7)	< 0.01
Gestational diabetes mellitus	73 (8.2)	27 (4.9)	100 (6.9)	0.03
Gestational age at FGR diagnosis (weeks)	28 + 5 (26 + 1 to 30 + 2)	26 + 4 (24 + 2 to 28 + 1)	27 + 6 (25 + 0 to 29 + 5)	< 0.01
Gestational age at admission (weeks)	30 + 0 ± 3 + 0	27 + 1 ± 2 + 0	28 + 6 ± 3 + 0	< 0.01
Use of antihypertensive drugs	247 (27.6)	210 (37.4)	457 (31.5)	< 0.01
Use of acetylsalicylic acid	101 (11.3)	85 (15.1)	186 (12.8)	0.03
Use of magnesium sulfate	145 (16.3)	154 (27.6)	299 (20.6)	< 0.01
Use of antenatal CCS at any time during pregnancy	756 (84.7)	541 (96.6)	1297 (89.3)	< 0.01
Ultrasound parameters				
Estimated fetal weight (g)	1120 ± 458	793 ± 376	993 ± 457	< 0.01
UA‐PI percentile	93.8 (59.7–99.8)	98.7 (80.1–100.0)	96.6 (66.7–100.0)	< 0.01
MCA‐PI percentile	5.1 (0.2–48.9)	1.24 (0.1–16.8)	2.99 (0.1–31.4)	< 0.01
UA‐A/REDF	180 (20.1)	216 (38.6)	395 (27.2)	< 0.01
Cerebroplacental ratio percentile	2.3 (0.1–22.3)	1.50 (0.2–10.4)	1.85 (0.1–18.0)	< 0.01

Data are given as mean ± SD, median (interquartile range) or *n* (%).

All counts, column totals and percentages are pooled across the 165 multiple imputed datasets.

* *P*‐values provided for one of the imputed datasets.

CAPO was defined as one or more of the following: intraventricular hemorrhage ≥ Grade III or venous infarction; culture‐proven sepsis; necrotizing enterocolitis ≥ Stage IIA; cystic periventricular leukomalacia; moderate or severe bronchopulmonary dysplasia; or perinatal or in‐hospital mortality.

A/REDF, absent or reversed end‐diastolic flow; APS, antiphospholipid syndrome; BMI, body mass index; CCS, corticosteroids; FGR, fetal growth restriction; HELLP, Hemolysis, Elevated Liver enzymes, and Low Platelet; MCA, middle cerebral artery; PE, pre‐eclampsia; PI, pulsatility index; PIH, pregnancy‐induced hypertension; SLE, systemic lupus erythematosus; UA, umbilical artery.

**Table 3:** Delivery and neonatal characteristics in pregnancies with and those without composite adverse perinatal outcome (CAPO), after multiple imputation

Parameter	Without CAPO (*n* = 893)	With CAPO (*n* = 560)	Overall (*n* = 1453)	*P* *
Timing of delivery				
Spontaneous	69 (7.8)	26 (4.6)	95 (6.5)	< 0.01
Iatrogenic	823 (92.2)	535 (95.4)	1358 (93.5)	< 0.01
Mode of delivery				
Vaginal	150 (16.8)	38 (6.7)	188 (12.9)	< 0.01
Prelabor Cesarean	643 (72.0)	511 (91.2)	1154 (79.4)	< 0.01
Emergency Cesarean	99 (11.1)	12 (2.1)	111 (7.6)	< 0.01
Gestational age at birth (weeks)	31 + 5 (30 + 2 to 34 + 2)	28 + 2 (27 + 0 to 29 + 5)	30 + 4 (28 + 4 to 32 + 3)	< 0.01
Birth weight (g)	1210 (1005–1510)	785 (630–985)	1050 (800–1345)	< 0.01
Male sex	379 (42.4)	288 (51.5)	667 (45.9)	< 0.01
CAPO	0 (0)	560 (100)	560 (38.5)	—
Perinatal mortality	0 (0)	55 (9.8)	55 (3.8)	—
In‐hospital mortality	0 (0)	106 (18.9)	106 (7.3)	—
Necrotizing enterocolitis ≥ Grade IIA	0 (0)	76 (13.6)	76 (5.2)	—
Cystic periventricular leukomalacia	0 (0)	10 (1.8)	10 (0.7)	—
Bronchopulmonary dysplasia	0 (0)	303 (54.1)	303 (20.9)	—
Intraventricular hemorrhage ≥ Grade III or venous infarction	0 (0)	26 (4.6)	26 (1.8)	—
Culture‐proven sepsis	0 (0)	267 (47.7)	267 (18.4)	—

Data are given as *n* (%) or median (interquartile range).

All counts, column totals and percentages are pooled across the 165 multiple imputed datasets.

* *P*‐values provided for one of the imputed datasets.

CAPO was defined as one or more of the following: intraventricular hemorrhage ≥ Grade III or venous infarction; culture‐proven sepsis; necrotizing enterocolitis ≥ Stage IIA; cystic periventricular leukomalacia; moderate or severe bronchopulmonary dysplasia; or perinatal or in‐hospital mortality.

### Model development

**Figure 1:**
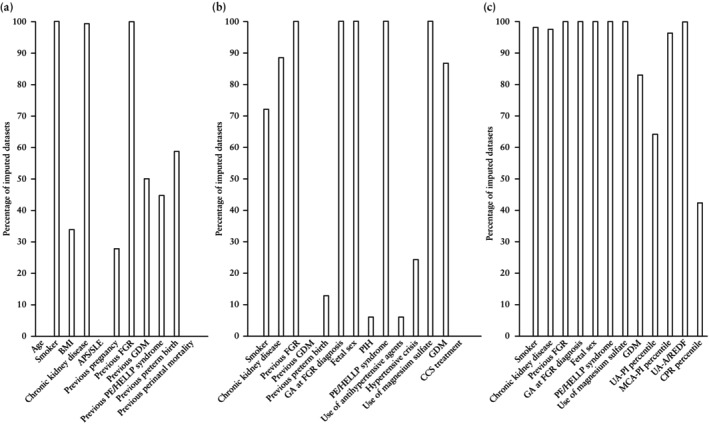
Model development across the 165 imputed datasets showing selection of predictors using backward stepwise selection in three phases: (a) Phase A, parameters with regard to baseline characteristics, medical history and obstetric history; (b) Phase B, parameters with regard to current pregnancy; and (c) Phase C, fetal ultrasound measurements. A/REDF, absent or reversed end‐diastolic flow; APS, antiphospholipid syndrome; BMI, body mass index; CCS, corticosteroids; CPR, cerebroplacental ratio; FGR, fetal growth restriction; GA, gestational age; GDM, gestational diabetes mellitus; HELLP, Hemolysis, Elevated Liver enzymes, and Low Platelet; MCA, middle cerebral artery; PE, pre‐eclampsia; PI, pulsatility index; PIH, pregnancy‐induced hypertension; SLE, systemic lupus erythematosus; UA, umbilical artery.

Model development was performed within each of the 165 imputed datasets and is summarized in Figure 1. Of the 27 candidate predictors, 14 were included in the final model. The regression formula was built using the data provided in Table 4, both before and after internal validation.

**Table 4:** Characteristics of final prediction model for composite adverse perinatal outcome (CAPO) at hospital admission for fetal monitoring in early‐onset fetal growth restriction (FGR)

Coefficient	Before internal validation	After internal validation†	Transformation formula
Intercept	11.777	10.819	N/A
EFW (in g)	−0.129	–0.119	((EFW/1000)ˆ2) + ((EFW/1000)ˆ2 * ln((EFW/1000)))
GA at admission (in days)	–0.062	–0.057	N/A
Chronic hypertension	–0.059	–0.054	N/A
Smoker	–0.357	–0.328	N/A
Chronic kidney disease	–0.664	–0.611	N/A
Previous pregnancy complicated by FGR	0.553	0.509	N/A
GA at FGR diagnosis (in days)	–0.121	–0.112	((GA at FGR diagnosis/100)ˆ2) + ((GA at FGR diagnosis/100)ˆ2 * ln((GA at FGR diagnosis/100)))
Fetal sex (male = 1, female = 0)	0.344	0.316	N/A
PE/HELLP syndrome	0.443	0.407	N/A
Use of magnesium sulfate	0.591	0.543	N/A
Gestational diabetes mellitus	–0.430	–0.396	N/A
UA‐PI percentile	0.239	0.220	((UA‐PI percentile/100)ˆ0.5) + ((UA‐PI percentile/100)ˆ1)
MCA‐PI percentile	–0.008	–0.007	N/A
UA‐A/REDF	0.683	0.628	N/A

Data are given as pooled estimate.

†Shrinkage factor = 0.920.

CAPO was defined as one or more of the following: intraventricular hemorrhage ≥ Grade III or venous infarction; culture‐proven sepsis; necrotizing enterocolitis ≥ Stage IIA; cystic periventricular leukomalacia; moderate or severe bronchopulmonary dysplasia; or perinatal or in‐hospital mortality.

A/REDF, absent or reversed end‐diastolic flow; EFW, estimated fetal weight; GA, gestational age; HELLP, Hemolysis, Elevated Liver enzymes, and Low Platelet; MCA, middle cerebral artery; N/A, not applicable; PE, pre‐eclampsia; PI, pulsatility index; UA, umbilical artery.

The risk of CAPO can be calculated using the formula 1/(1 + exp^−linear predictor^), in which the linear predictor, after internal validation, equals: 10.819 + (transformed EFW × −0.119) + (GA at admission (days) × −0.057) + (chronic hypertension × −0.054) + (smoker × −0.328) + (chronic kidney disease × −0.611) + (previous pregnancy complicated by FGR × 0.509) + (transformed GA at FGR diagnosis (days) × −0.112) + (fetal sex (male = 1, female = 0) × 0.316) + (PE/HELLP syndrome at admission × 0.407) + (use of magnesium sulfate at admission × 0.543) + (gestational diabetes mellitus × −0.396) + (transformed UA‐PI percentile × 0.220) + (MCA‐PI percentile × −0.007) + (UA‐A/REDF × 0.628), where EFW is estimated fetal weight and GA is gestational age. Transformations are given in Table 4. Examples of early‐onset FGR cases along with the calculated risk of CAPO are provided in Table S5.

### Model performance

**Figure 2:**
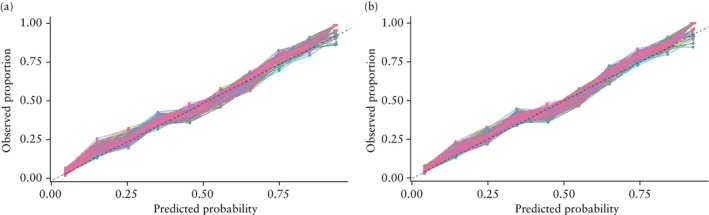
Calibration plots before (a) and after (b) internal validation for the developed model (for all 165 datasets). 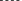 represents perfect calibration.

Visual inspection of the calibration plots revealed good model calibration both before and after internal validation (Figures 2, S1 and S2). After internal validation and shrinkage of the coefficients, the AUC was 0.83 (95% CI, 0.79–0.87), the calibration slope was 1.05 (95% CI, 0.94–1.17) and calibration‐in‐the‐large was 0.07 (95% CI, −0.06 to 0.20) (Table 5).

**Table 5:** Performance of final prediction model for composite adverse perinatal outcome (CAPO) at hospital admission for fetal monitoring in early‐onset fetal growth restriction

Performance measure	Before internal validation	After internal validation
Model discrimination		
AUC	0.83 (0.79 to 0.87)	0.83 (0.79 to 0.87)
Model calibration		
Calibration slope	0.97 (0.86 to 1.08)	1.05 (0.94 to 1.17)
Calibration‐in‐the‐large	0.08 (−0.05 to 0.21)	0.07 (−0.06 to 0.20)

Data are given as pooled estimate (95% CI).

CAPO was defined as one or more of the following: intraventricular hemorrhage ≥ Grade III or venous infarction; culture‐proven sepsis; necrotizing enterocolitis ≥ Stage IIA; cystic periventricular leukomalacia; moderate or severe bronchopulmonary dysplasia; or perinatal or in‐hospital mortality.

AUC, area under the receiver‐operating‐characteristics curve.

### Internal–external cross‐validation

**Figure 3:**
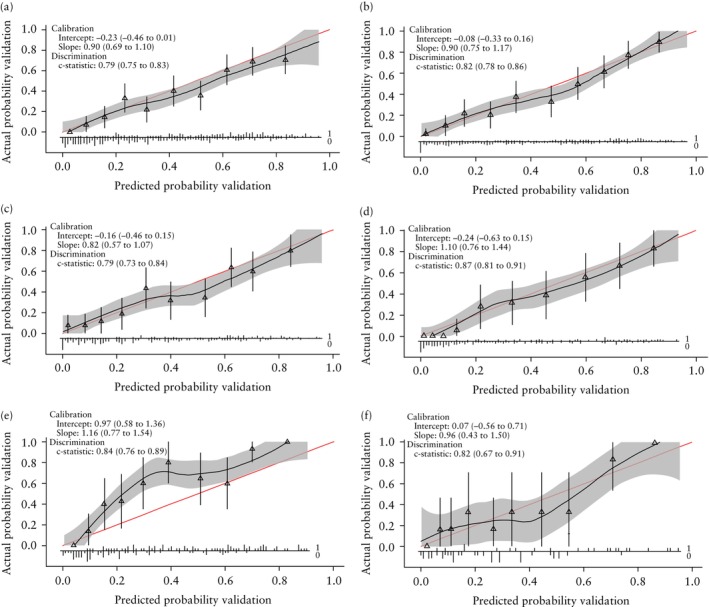
Calibration plots following internal–external cross‐validation for the developed model (provided for one of the imputed datasets), with external validation performed in: (a) University Medical Center Utrecht (*n* = 416); (b) Erasmus Medical Center (*n* = 395); (c) Amsterdam University Medical Center (*n* = 253); (d) Maastricht University Medical Center (*n* = 182); (e) University Medical Center Groningen (*n* = 147); and (f) Isala Hospital, Zwolle (*n* = 60). Red line represents perfect calibration. Shaded area represents 95% CI and triangles represent groups ranked by estimated probability.

**Figure 4:**
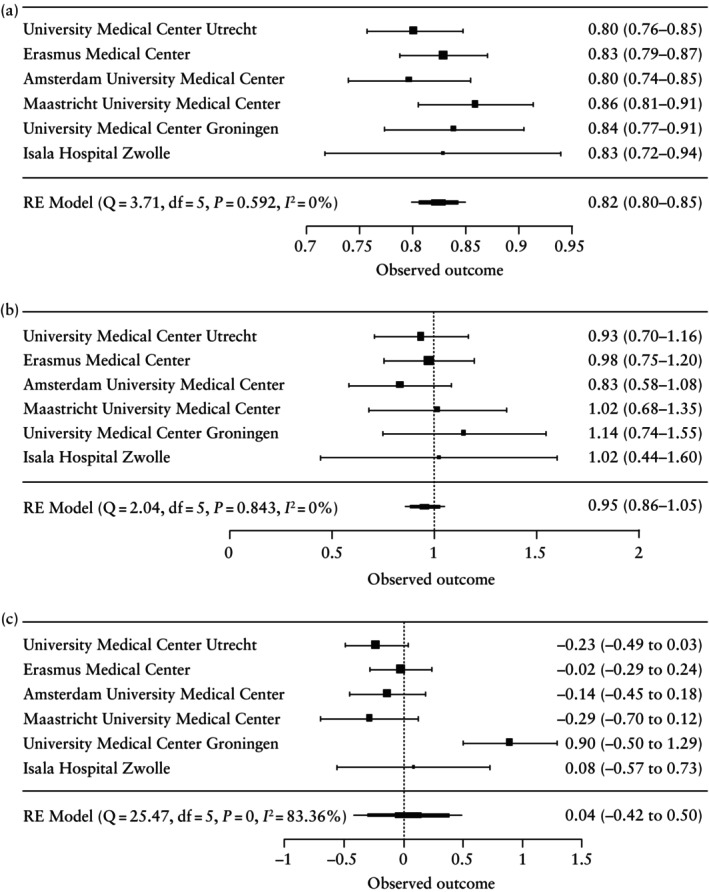
Forest plots of model performance across hospitals after internal–external cross‐validation (each hospital serving as external validation dataset once), showing: (a) area under receiver‐operating‐characteristics curve; (b) calibration slope; and (c) calibration‐in‐the‐large. Performance measures are presented with 95% CIs. *P*‐values test for heterogeneity. df, degrees of freedom; RE, random effects.

Internal–external cross‐validation of the model, including the final predictors, was performed as a second step to assess the robustness of the predictive properties of the model. Calibration plots, in which each hospital served as the external validation cohort once, are shown for 1/165 imputed datasets (Figure 3). Overall, on visual inspection of the calibration plots, model calibration was good across hospitals, except for the two hospitals with the smallest sample sizes (Maastricht University Medical Center and University Medical Center Groningen). Meta‐analysis of model performance across hospitals revealed that the AUC was 0.82 (95% CI, 0.80–0.85), the calibration slope was 0.95 (95% CI, 0.86–1.05) and calibration‐in‐the‐large was 0.04 (95% CI, −0.42 to 0.50) (Table S6). Model performance was consistent across hospitals for the AUC and calibration slope (*I* ^2^ = 0% for both), but heterogeneous for the calibration‐in‐the‐large (*I* ^2^ = 83.4%). Heterogeneity for calibration‐in‐the‐large was primarily the result of a divergent estimate from the external validation in the University Medical Center Groningen, which may be explained by the small sample size of patients included from this hospital (Figure 4). No heterogeneity was seen for the calibration‐in‐the‐large when internal–external cross‐validation analyses were performed only in the three largest hospitals (University Medical Center Utrecht, Erasmus Medical Center and Amsterdam University Medical Center) (*I* ^2^ = 0%).

## DISCUSSION

In this study, a prediction model was developed and internally validated using readily available predictors for CAPO in early‐onset FGR. The developed model shows a good ability to discriminate between pregnancies with and those without CAPO, reflected by an AUC of 0.83 (95% CI, 0.79–0.87). Furthermore, good calibration was reflected by a calibration slope of 1.05 (95% CI, 0.94–1.17) and calibration‐in‐the‐large of 0.07 (95% CI, −0.06 to 0.20). Additionally, internal–external cross‐validation showed that, overall, model performance was stable across the four hospitals with the largest sample sizes, suggestive of a robust predictive performance of the developed model.

We have developed a new prediction model to be used at hospital admission for fetal monitoring in pregnancies diagnosed with early‐onset FGR. Guidelines for prediction‐modeling studies recommend externally validating or updating existing models, instead of continuously creating new models^35^. However, none of the 11 previously developed models were developed specifically for prediction at hospital admission (rather 24–72 h before delivery) in early‐onset FGR^8^, ^12^, ^36^, ^37^, ^38^, ^39^, ^40^, ^41^, ^42^, ^43^, ^44^, ^45^. In addition, the definition and predicted outcomes of early‐onset FGR vary across studies, limiting external validation within our cohort^12^. Three existing models included biomarkers that are not obtained in routine clinical practice in most countries and that were not measured in our cohort^8^, ^38^, ^39^. Lastly, model performance measures and data required for external validation were lacking in most existing models, further hampering the possibility of performing external validation of these models in our cohort^12^. The number of drawbacks of the existing models led to the decision to create a new model, rather than updating an existing model^4^, ^15^. Although comparing performance across FGR prediction models is challenging owing to variations in the timing of prediction and variations in FGR and outcome definitions, our model's performance was comparable with, or even superior to, previously developed models (AUC reported in 5/10 models ranging from 0.64–0.90; calibration slope reported for models from only one study ranging from 0.78–0.99)^8^, ^36^, ^38^, ^39^, ^44^, ^45^. Remarkably, smoking appears to be protective against CAPO, but this is probably due to higher gestational ages at birth in pregnancies among smokers (mean, 219 ± 23 days *vs* 215 ± 22 days; *P* < 0.01). Additionally, chronic hypertension and chronic kidney disease appear protective against CAPO in the multivariate prediction model. However, these findings should be viewed in the context of the multivariable regression prediction model, emphasizing that the protective effects of smoking, chronic hypertension and chronic kidney disease should not be interpreted as causal.

### Clinical implications

The developed model could provide individual risk assessments of CAPO in early‐onset FGR pregnancies. This could aid clinical decision‐making, as shown inTable 5, and parental expectation management following birth, which is often preterm. It could also aid parental counseling by providing a risk assessment of CAPO, which could help parents decide whether to consent to active fetal, and thus neonatal, management (as shown in Table 5). Nevertheless, prior to the adoption of the model in clinical practice, it should be externally validated and, if necessary, updated within another cohort, and thereafter assessed for its impact on clinical decision‐making and patient outcomes^43^.

### Research implications

The next step would be to conduct external validation of the developed model to assess its robustness across different populations^46^. Additionally, a multivariable dynamic prediction tool would be even more beneficial than a static prediction model, as it can generate daily updates on the risk of perinatal complications using newly derived clinical information on fetal and maternal health status. This approach better aligns with clinical practice, in which obstetricians use updated parameter measurements on a day‐to‐day basis for clinical decision‐making (e.g. for the timing and healthcare setting of birth). Future research should therefore focus on the development of this methodology and, consequently, the development of a dynamic prediction tool in the setting of early‐onset FGR.

### Strengths and limitations

The strengths of this study are: (1) the large dataset used for model development; (2) the use of a consensus‐based definition (Gordijn *et al*.^15^) of the population and outcome in accordance with a landmark trial in early‐onset FGR (the TRUFFLE study^4^); (3) the reporting of the development and internal validation of the model in accordance with the TRIPOD guidelines^17^; (4) the state‐of‐the‐art methodology used for model development and internal validation; (5) cross‐checking the neonatal outcome data with the Dutch birth registry^16^; and (6) the performance of internal–external cross‐validation to account for the clustering of patients within each hospital.

The study was limited by its retrospective design, which made it prone to loss‐to‐follow‐up for data concerning long‐term neurodevelopmental outcomes, which would have been an alternative, interesting outcome for the prediction model^47^. Additionally, ductus venosus measurements were excluded owing to 88% missing values; adding this as a predictor might have improved the model's performance. However, this reflects real‐world data, where the use of ductus venosus measurements is limited in some countries. The already high predictive value of the model makes it usable in clinical practice, even in settings without reliable ductus venosus measurements. Furthermore, the study lacked external validation, which is an important step that should be undertaken before implementation of the model in clinical practice. Additionally, the indications for hospital admission for fetal surveillance differ between countries, which may limit application of the model in other countries and settings. Therefore, external validation, and updating the model if necessary, are essential. Moreover, implementation of the model risks creating a self‐fulfilling prophecy, so it is therefore important to highlight these limitations during interpretation and implementation. Lastly, as patients were only included if they consented to active fetal management, the model is not suitable for providing risk estimates of CAPO when parents do not consent to active fetal management.

### Conclusions

This study provides a prediction model, including 14 readily available predictors, which could serve as a risk‐assessment tool during parental counseling in pregnancies complicated by early‐onset FGR, and aid the clinical decision‐making of physicians when a gestational age > 24 weeks has been reached. Future research should focus on external validation of the model to test its robustness in other hospitals and countries for managing early‐onset FGR pregnancies and, thereafter, assessment of its impact on clinical decision‐making and patient outcomes.

## Supporting information


**Appendix S1** TRIPOD Checklist: Prediction Model Development
**Table S1** Definitions of adverse perinatal outcomes
**Table S2** Demographic and baseline characteristics at admission, with percentages of missing data (before multiple imputation)
**Table S3** Delivery and neonatal outcome data, with percentages of missing data (before multiple imputation)
**Table S4** Univariable regression analysis of association between candidate predictors and composite adverse perinatal outcome
**Table S5** Examples of early‐onset fetal growth restriction (FGR) cases
**Table S6** Meta‐analysis of model performance following internal–external cross‐validation
**Figure S1** Calibration plot in 1/165 imputed datasets, before internal validation.
**Figure S2** Calibration plot in 1/165 imputed datasets, after internal validation.

## Data Availability

The data that support the findings of this study are available from the corresponding author upon reasonable request.
