# Supplementary material for: Perinatal risk assessment in pregnancies complicated by early‐onset fetal growth restriction: development and internal validation of a prediction model for composite adverse perinatal outcome
Source: Ultrasound Obstet Gynecol. 2025 Jul 7;66(2):175–85. doi: 10.1002/uog.29265 (PMC12317299; doi:10.1002/uog.29265)
Supplement: Supplementary file 1 — Appendix S1 TRIPOD Checklist: Prediction Model Development Table S1 Definitions of adverse perinatal outcomes Table S2 Demographic and baseline characteristics at admission, with percentages of missing data (before multiple imputation) Table S3 Delivery and neonatal outcome data, with percentages of missing data (before multiple imputation) Table S4 Univariable regression analysis of association between candidate predictors and composite adverse perinatal outcome Table S5 Examples of early‐onset fetal growth restriction (FGR) cases Table S6 Meta‐analysis of model performance following internal–external cross‐validation Figure S1 Calibration plot in 1/165 imputed datasets, before internal validation. Figure S2 Calibration plot in 1/165 imputed datasets, after internal validation. [file UOG-66-175-s001.docx]

**SUPPLEMENTARY INFORMATION FOR REVIEW**

**Appendix S1.** TRIPOD Checklist: Prediction Model Development

| **Section/Topic Item Checklist Item Page** | | | |
| --- | --- | --- | --- |
| **Title and abstract** | | | |
| Title | 1 | Identify the study as developing and/or validating a multivariable prediction model, the target population, and the outcome to be predicted. | 1 |
| Abstract | 2 | Provide a summary of objectives, study design, setting, participants, sample size, predictors, outcome, statistical analysis, results, and conclusions. | 2-3 |
| **Introduction** | | | |
| Background and objectives | 3a | Explain the medical context (including whether diagnostic or prognostic) and rationale for developing or validating the multivariable prediction model, including references to existing models. | 4 |
|  | 3b | Specify the objectives, including whether the study describes the development or validation of the model or both. | 5 |
| **Methods** | | | |
| Source of data | 4a | Describe the study design or source of data (e.g., randomized trial, cohort, or  registry data), separately for the development and validation data sets, if applicable. | 5 |
|  | 4b | Specify the key study dates, including start of accrual; end of accrual; and, if applicable, end of follow-up. | 5 |
| Participants | 5a | Specify key elements of the study setting (e.g., primary care, secondary care, general population) including number and location of centres. | 5 |
|  | 5b | Describe eligibility criteria for participants. | 5 |
|  | 5c | Give details of treatments received, if relevant. | 5 |
| Outcome | 6a | Clearly define the outcome that is predicted by the prediction model, including how and when assessed. | 6 |
|  | 6b | Report any actions to blind assessment of the outcome to be predicted. | NA |
| Predictors | 7a | Clearly define all predictors used in developing or validating the multivariable  prediction model, including how and when they were measured. | 5 |
|  | 7b | Report any actions to blind assessment of predictors for the outcome and other predictors. | NA |
| Sample size | 8 | Explain how the study size was arrived at. | 6 |
| Missing data | 9 | Describe how missing data were handled (e.g., complete-case analysis, single imputation, multiple imputation) with details of any imputation method. | 6 |
| Statistical analysis methods | 10a | Describe how predictors were handled in the analyses. | 7,8 |
|  | 10b | Specify type of model, all model-building procedures (including any predictor selection), and method for internal validation. | 7,8 |
|  | 10d | Specify all measures used to assess model performance and, if relevant, to compare multiple models. | 7,8 |
| Risk groups | 11 | Provide details on how risk groups were created, if done. | NA |
| **Results** | | | |
| Participants | 13a | Describe the flow of participants through the study, including the number of participants with and without the outcome and, if applicable, a summary of the follow-up time. A diagram may be helpful. | 9 |
|  | 13b | Describe the characteristics of the participants (basic demographics, clinical features, available predictors), including the number of participants with missing  data for predictors and outcome. | 9, 20-22, Table S2-S3 |
| Model development | 14a | Specify the number of participants and outcome events in each analysis. | 9, 22, Figure S2 |
|  | 14b | If done, report the unadjusted association between each candidate predictor and outcome. | 9, Table S4 |
| Model specification | 15a | Present the full prediction model to allow predictions for individuals (i.e., all regression coefficients, and model intercept or baseline survival at a given time  point). | 23 |
|  | 15b | Explain how to the use the prediction model. | 10 |
| Model performance | 16 | Report performance measures (with CIs) for the prediction model. | 9-10, Figure 2-4 |
| **Discussion** | | | |
| Limitations | 18 | Discuss any limitations of the study (such as nonrepresentative sample, few events  per predictor, missing data). | 13 |
| Interpretation | 19b | Give an overall interpretation of the results, considering objectives, limitations, and  results from similar studies, and other relevant evidence. | 10-13 |
| Implications | 20 | Discuss the potential clinical use of the model and implications for future research. | 11, 12 |
| **Other information** | | | |
| Supplementary information | 21 | Provide information about the availability of supplementary resources, such as study protocol, Web calculator, and data sets. | Supplementary information file |
| Funding | 22 | Give the source of funding and the role of the funders for the present study. | 14 |

**Table S1.** Definitions of adverse perinatal outcomes

| Outcome | Definition |
| --- | --- |
| Primary outcome |  |
| Perinatal mortality | Perinatal mortality was defined as death from 22 completed weeks of gestation up to seven days following birth and in-hospital mortality from birth to home discharge of the infant^45^ |
| Infant mortality until hospital discharge | Death of the infant until hospital-discharge |
| Secondary outcome measures |  |
| Early-onset sepsis (culture-proven) | Culture-proven, neonatal sepsis in the first 72 hours of age |
| Late-onset sepsis (culture-proven) | Culture-proven, neonatal sepsis after the first 72 hours of age |
| Necrotizing enterocolitis Bell’s Stage ≥ 2A | Definitive medical necrotizing enterocolitis:   - Abdominal distention with pneumatosis intestinalis, portal venous gas, or both - Other radiographic signs such as fixed, dilated loops of intestine and ileus patterns are not pathognomonic but should be treated as such   Surgical necrotizing enterocolitis:   - Free intraperitoneal air on abdominal radiograph after initial medical signs and symptoms - Persistent ileus pattern, abdominal distension, and radiographs that show an absence of bowel gas, coupled with deteriorating clinical and laboratory values^46^ |
| Intraventricular hemorrhage grade ≥ 3 | Intraventricular hemorrhage grade 3 according to Papile et al., venous infarction, posthemorrhagic ventricular dilatation needing treatment^47^ |
| Cystic periventricular leukomalacia | Cystic periventricular leukomalacia characterized by diffuse injury of the white matter, which possibly leads to cerebral palsy^48^ |
| Bronchopulmonary dysplasia, moderate and severe | Bronchopulmonary dysplasia is diagnosed if gestational age <32 weeks: at a postmenstrual age of 36 weeks, >21% oxygen has been administered cumulatively for 28 or more days^49^   - Moderate: Need for <30% oxygen at 36 weeks postmenstrual age - Severe: Need for ≥30% oxygen and/or positive pressure (positive pressure ventilation or continuous positive airway pressure) at 36 weeks postmenstrual age |

**Table S2.** Demographic and baseline characteristics at admission with percentages of missing data (before multiple imputation)

|  | Overall  (N=1453) |
| --- | --- |
| **Demographic** | |
| Age | 30.6 (5.45) |
| BMI  *Missing* | 24.6 (21.5, 29.1) *310 (21.3)* |
| Caucasian ethnicity *Missing* | 905 (62.3) *209 (14.4)* |
| **Medical history** | |
| Chronic hypertension | 203 (14.0) |
| Chronic kidney disease | 47 (3.3) |
| Antiphospholipid syndrome/systemic lupus erythematosus | 16 (1.1) |
| Pre-existent diabetes mellitus | 32 (2.2) |
| Smoking during pregnancy *Missing* | 227 (15.6) *18 (1.2)* |
| **Obstetric history** | |
| Previous pregnancy   - Previous FGR   *Missing*   - Previous PIH *Missing* - Previous PE/HELLP *Missing* - Previous preterm birth *Missing* - Perinatal mortality in previous pregnancy   *Missing*   - Previous diabetes gravidarum   *Missing* | 525 (36.1)   - 155 (10.7) *32 (2.2)* - 36 (2.5)   *29 (2.0)*   - 118 (8.1) *29 (2.0)* - 206 (14.2)   *10 (0.7)*   - 65 (4.5) *6 (0.4)* - 21 (1.4)   *35 (2.4)* |
| **Current pregnancy** | |
| PIH *Missing* | 169 (11.6)  104 (7.2) |
| PE/HELLP *Missing* | 451 (31.0) 104 (7.2) |
| Diabetes gravidarum | 100 (6.9) |
| Gestational age at FGR diagnosis (weeks) | 27 ⁶/₇ (25 ⁰/₇, 29 ⁵/₇) |
| Gestational age at admission (weeks) | 28 ⁶/₇ (3.0) |
| **Medication use in current pregnancy** | |
| Use of antihypertensive agents *Missing* | 398 (27.4) *106 (7.3)* |
| Use of acetylsalicylic acid | 186 (12.8) |
| Use of magnesiumsulfate *Missing* | 227 (15.6) *106 (7.3)* |
| Administration of antenatal CCS | 1297 (89.3) |
| **Ultrasound parameters** | |
| Estimated fetal weight (grams)  *Missing* | 956 (365.6) *104 (7.2)* |
| Percentile PI UA  *Missing* | 96.7 (78.1, 99.9)  *247 (17.0)* |
| Percentile PI MCA  *Missing* | 3.1 (0.30, 19.2)  *391 (27.0)* |
| Absent or reversed EDF  *Missing* | 318 (21.9) *181 (12.5)* |
| Cerebroplacental ratio  *Missing* | 1.02 (0.73, 1.35) *431 (30.0)* |

Data provided as mean (SD), median (IQR) or N (%) as appropriate. Abbreviations: BMI, body mass index; CCS, corticosteroids; EDF, end-diastolic flow; FGR, fetal growth restriction; HELLP, hemolysis elevated liver enzymes low platelets; MCA, middle cerebral artery; PE, pre-eclampsia; PI, pulsatility index; PIH, pregnancy-induced hypertension; UA, umbilical artery.

**Table S3.** Delivery and neonatal outcome data with percentages of missing data (before multiple imputation)

|  | Overall  (N=1453) |
| --- | --- |
| **Delivery** | |
| Gestational age at delivery | 30 ⁴/₇ (28 ⁴/₇ , 32 ³/₇) |
| Timing of delivery   - Spontaneous - Iatrogenic | 95 (6.5) 1357 (93.5) |
| Indication iatrogenic delivery   - Maternal indication - Fetal indication - Maternal and fetal indication - Other | 252 (17.4) 966 (66.5) 129 (8.9) 10 (0.7) |
| Mode of delivery   - Vaginally - Prelabour section - Emergency section | 188 (12.9) 1153 (79.4) 111 (7.6) |
| **Neonatal outcomes** | |
| Birthweight | 1050 (800, 1345) |
| Male sex | 667 (45.9) |
| Adverse perinatal outcomes   - Perinatal mortality - Infant, in-hospital mortality - Necrotizing enterocolitis ≥2A - Cystic periventricular leukomalacia - Bronchopulmonary dysplasia   *Missing*   - Intraventricular hemorrhage ≥grade 3 - Culture-proven sepsis | - 55 (3.8) - 106 (7.3) - 76 (5.2) - 10 (0.7) - 266 (18.3)   *89 (6.1)*   - 26 (1.8) - 288 (19.8) |

Data provided as median (IQR), mean (SD) or N (%) as appropriate.

**Table S4.** Univariable regression analysis of association between candidate predictors and composite adverse perinatal outcome

| Candidate predictor | OR (95% CI) | Transformation (if required) |
| --- | --- | --- |
| Phase A: Baseline characteristics, medical and obstetric history | | |
| Age | 1.00 (0.98-1.02) |  |
| BMI | 1.01 (1.00-1.02) |  |
| Smoking | 0.66 (0.49-0.89) |  |
| Chronic hypertension | 1.68 (1.25-2.27) |  |
| Chronic kidney disease | 1.14 (0.63-2.06) |  |
| APS/SLE | 1.60 (0.60-4.29) |  |
| Previous pregnancy (multipara) | 0.94 (0.76-1.18) |  |
| Previous FGR | 1.36 (0.98-1.89) |  |
| Previous preterm birth | 1.18 (0.88-1.59) |  |
| Previous PE/HELLP | 1.69 (1.17-2.46) |  |
| Previous perinatal mortality | 1.04 (0.63-1.73) |  |
| Previous diabetes gravidarum | 0.72 (0.31-1.69) |  |
| Phase B: Current pregnancy | | |
| Gestational age at FGR diagnosis (days) | 0.73 (0.69-0.78) | ((GA at FGR diagnosis/100)^2)+((GA at FGR diagnosis/100)^2*log((GA at FGR diagnosis/100))) |
| Sex neonate | 1.44 (1.16-1.78) |  |
| PIH diagnosis | 0.94 (0.70-1.26) |  |
| PE/HELLP diagnosis | 1.79 (1.44-2.22) |  |
| Use of antihypertensive drugs | 1.57 (1.25-1.96) |  |
| Hypertensive crise | 1.86 (1.38-2.50) |  |
| Use of magnesiumsulfate | 1.96 (1.52-2.54) |  |
| Diabetes gravidarum diagnosis | 0.57 (0.36-0.90) |  |
| Use of antenatal CCS | 5.16 (3.16-8.45) |  |
| Phase C: Ultrasound parameters | | |
| Percentile PI umbilical artery | 1.43 (1.17-1.74) | ((Percentile PI UA/100)^0.5)+(( Percentile PI UA/100)^1) |
| Percentile PI middle cerebral artery | 0.99 (0.99-0.99) |  |
| End-diastolic velocity umbilical artery | 2.52 (1.99-3.20) |  |
| Percentile cerebroplacental ratio | 0.66 (0.48-0.91) | ((Percentile CPR/100)^1)+((Percentile_CPR/100)^3) |
| Fixed parameters | | |
| Gestational age at admission | 0.93 (0.92-0.94) |  |
| Estimated fetal weight (grams) | 0.51 (0.46-0.58) | ((EFW/1000)^2)+((EFW/1000)^2*log((EFW/1000))) |

Abbreviations: APS, antiphospholipid syndrome; BMI, body mass index; CCS, corticosteroids; CPR, cerebroplacental ratio; EDF, end-diastolic flow; EFW, estimated fetal weight; FGR, fetal growth restriction; GA, gestational age; HELLP, hemolysis elevated liver enzymes low platelets; PE, pre-eclampsia; PI, pulsatility index; PIH, pregnancy-induced hypertension; UA, umbilical artery; SLE, systemic lupus erythematosus.

**Table S5** Examples of early-onset fetal growth restriction (FGR) cases

| Intended use of the model | Case description | Example calculation |
| --- | --- | --- |
| Parental counseling for active fetal, and thus neonatal, management | A woman in her first pregnancy, who is a non-smoker, with no notable medical history. She is diagnosed with early-onset FGR at 22 weeks and 4 days (158 days) of gestational age. At 24 weeks and 6 days (174 days) of gestational age the estimated fetal weight is 607 g. Doppler assessment shows absent end-diastolic velocity in the umbilical artery, a pulsatility index percentile of 99.97 in the umbilical artery, and a pulsatility index percentile of 0.26 in the middle cerebral artery. The fetal sex was determined to be female. She has been diagnosed with concomitant PE/HELLP syndrome, but not with gestational diabetes mellitus and is not treated with magnesium sulfate.  The consulting neonatologist and gynecologist counsel the patient regarding the risk of adverse neonatal outcomes using the prediction model. Based on a 87.5% risk of adverse neonatal outcomes, parents can decide whether they want to opt for admission with active fetal management. | - Transformation of continuous variables   ((607/1000)^2)+((607/1000)^2*ln((607/1000))) = 0.1845  ((158/100)^2) + ((158/100)^2 * ln((158/100))) = 3.638  ((99.97/100)^0.5)+((99.97/100)^1) = 2.000   - Linear predictor   10.819 + (0.1845*-0.119) + (174*-0.057) + (0*-0.054) + (0*-0.328) + (0*-0.611) + (0*0.509) + (3.638*-0.112) + (0*0.316) + (1*0.407) + (0*0.543) + (0*-0.396) + (2.000*0.220) + (0.26*-0.007) + (1*0.628) = 1.94   - Predicted risk of adverse perinatal outcome   1/(1+exp^-1.94^) = 0.8749 |
| Determining whether a patient with early-onset FGR needs to be referred to a hospital with a neonatal intensive care unit | A patient in her first pregnancy. She is a smoker and has no notable medical history. She is diagnosed with early-onset FGR at 29 weeks and 6 days (210 days) of gestational age.  At 30 weeks and 1 day (211 days) of gestational age she visits a level-II hospital. Ultrasound examination reveals an estimated fetal weight of 1050 grams, an abnormal pulsatility index of the umbilical artery, with a percentile of 97 and a positive end-diastolic velocity and a pulsatility index percentile of the middle cerebral artery of 6. The fetal sex was determined to be female. She is not diagnosed with concomitant PE/HELLP syndrome or gestational diabetes mellitus and is not treated with magnesium sulfate.  Based on the prediction model, the risk of adverse neonatal outcomes is 10.4% and the patient is transferred to a level-III hospital for follow-up and delivery. | - Transformation of continuous variables   ((1050/1000)^2)+((1050/1000)^2*ln((1050/1000))) = 1.156  ((210/100)^2) + ((210/100)^2 * ln((210/100))) = 7.682  ((97/100)^0.5)+((97/100)^1) = 1.955   - Linear predictor   10.819 + (1.156*-0.119) + (211*-0.057) + (0*-0.054) + (1*-0.328) + (0*-0.611) + (0*0.509) + (7.682*-0.112) + (0*0.316) + (0*0.407) + (0*0.543) + (0*-0.396) + (1.955*0.220) + (6*-0.007) + (0*0.628) =  -2.15   - Predicted risk of adverse perinatal outcome   1/(1+exp^---2.15)^) = 0.104 |

PE, pre-eclampsia.

**Table S6** Meta-analysis of model performance following internal–external cross-validation

| Performance statistic | Summary | Lower CI | Upper CI | Lower PI | Upper PI | Heterogeneity (I²) |
| --- | --- | --- | --- | --- | --- | --- |
| *Model discrimination*  AUC | 0.82 | 0.80 | 0.85 | 0.80 | 0.85 | 0.00% |
| *Model calibration*  Calibration slope  Calibration-in-the-large | 0.95  0.04 | 0.86  -0.42 | 1.05  0.50 | 0.86  -1.07 | 1.05  1.15 | 0.00%  83.36% |

AUC, area under receiver-operating-characteristics curve; PI, prediction interval.

**Figure S1.** Calibration plot in 1/165 imputed datasets, before internal validation


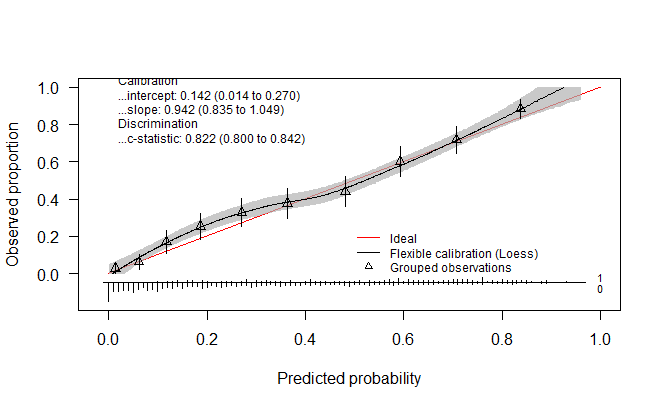


Patients were ranked by estimated probability, divided in ten groups and subsequently the average predicted probability and average observed outcome was determined. The red, 45-degree line represents perfect calibration.

**Figure S2.** Calibration plot in 1/165 imputed datasets, after internal validation

**
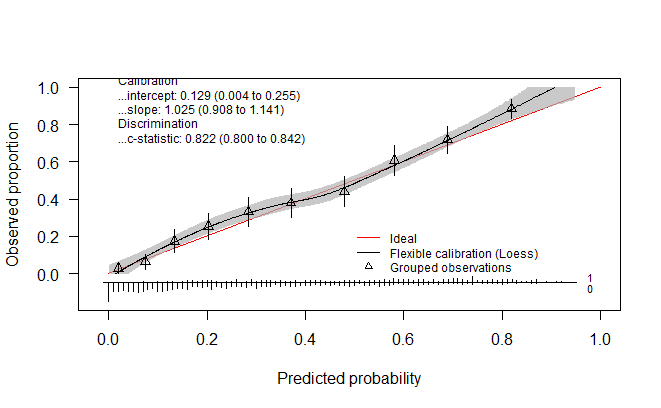
**

Patients were ranked by estimated probability, divided in ten groups and subsequently the average predicted probability and average observed outcome was determined. The red, 45-degree line represents perfect calibration.
